# Machine learning-based analysis of the impact of 5′ untranslated region on protein expression

**DOI:** 10.1093/nar/gkaf861

**Published:** 2025-09-09

**Authors:** Linfeng Wang, Sujia Liu, Jia-xin Huang, Haifeng Zhu, Shuyu Li, Yannan Li, Sen Chen, Jianying Han, Yin Zhu, Jiahao Wu, Wentao Liao, Hongmei Zhang, Haiyan Zeng, Shaoting Li, Shuping Zhao, Bingwei Wang, Jiaqi Lin, Ji Zeng

**Affiliations:** School of Biomedical and Pharmaceutical Sciences, Guangdong University of Technology, No. 100 Waihuanxi Road, Guangzhou 510006, China; MOE Key Laboratory of Bio-Intelligent Manufacturing, School of Bioengineering, Dalian University of Technology, Dalian 116024, China; Department of Liver Surgery, State Key Laboratory of Oncology in South China, Guangdong Provincial Clinical Research Center for Cancer, Collaborative Innovation Center for Cancer Medicine, Sun Yat-Sen University Cancer Center, Guangzhou 510060, China; School of Biomedical and Pharmaceutical Sciences, Guangdong University of Technology, No. 100 Waihuanxi Road, Guangzhou 510006, China; School of Biomedical and Pharmaceutical Sciences, Guangdong University of Technology, No. 100 Waihuanxi Road, Guangzhou 510006, China; School of Biomedical and Pharmaceutical Sciences, Guangdong University of Technology, No. 100 Waihuanxi Road, Guangzhou 510006, China; School of Biomedical and Pharmaceutical Sciences, Guangdong University of Technology, No. 100 Waihuanxi Road, Guangzhou 510006, China; School of Biomedical and Pharmaceutical Sciences, Guangdong University of Technology, No. 100 Waihuanxi Road, Guangzhou 510006, China; School of Biomedical and Pharmaceutical Sciences, Guangdong University of Technology, No. 100 Waihuanxi Road, Guangzhou 510006, China; School of Biomedical and Pharmaceutical Sciences, Guangdong University of Technology, No. 100 Waihuanxi Road, Guangzhou 510006, China; School of Biomedical and Pharmaceutical Sciences, Guangdong University of Technology, No. 100 Waihuanxi Road, Guangzhou 510006, China; School of Biomedical and Pharmaceutical Sciences, Guangdong University of Technology, No. 100 Waihuanxi Road, Guangzhou 510006, China; School of Biomedical and Pharmaceutical Sciences, Guangdong University of Technology, No. 100 Waihuanxi Road, Guangzhou 510006, China; School of Biomedical and Pharmaceutical Sciences, Guangdong University of Technology, No. 100 Waihuanxi Road, Guangzhou 510006, China; School of Biomedical and Pharmaceutical Sciences, Guangdong University of Technology, No. 100 Waihuanxi Road, Guangzhou 510006, China; Department of Urology, Guangdong Second Provincial General Hospital, No. 446 Xingang Middle Road, Guangzhou 510317, China; MOE Key Laboratory of Bio-Intelligent Manufacturing, School of Bioengineering, Dalian University of Technology, Dalian 116024, China; School of Biomedical and Pharmaceutical Sciences, Guangdong University of Technology, No. 100 Waihuanxi Road, Guangzhou 510006, China

## Abstract

The 5′ untranslated region (5′UTR) plays a crucial regulatory role in messenger RNA (mRNA), with modified 5′UTRs extensively utilized in vaccine production, gene therapy, etc. Nevertheless, manually optimizing 5′UTRs may encounter difficulties in balancing the effects of various *cis-*elements. Consequently, multiple 5′UTR libraries have been created, and machine learning models have been employed to analyze and predict translation efficiency (TE) and protein expression, providing insights into critical regulatory features. On the one hand, these screening libraries, based on TE and mean ribosome load, struggle to accurately quantify protein expression; on the other hand, a precise method for quantifying 5′UTRs necessitates a significantly costlier library. To resolve this dilemma, we constructed a library utilizing firefly luciferase as the reporter to measure accurate protein expression. In addition, we optimized the library construction method by clustering mRNA sequences to reduce redundant data and minimize the size of the dataset. This dual strategy by increasing accuracy and reducing dataset size was found to be effective in predicting the 5′UTRs from the PC3 cell line.

## Graphical Abstract

**Graphical Abstract:**
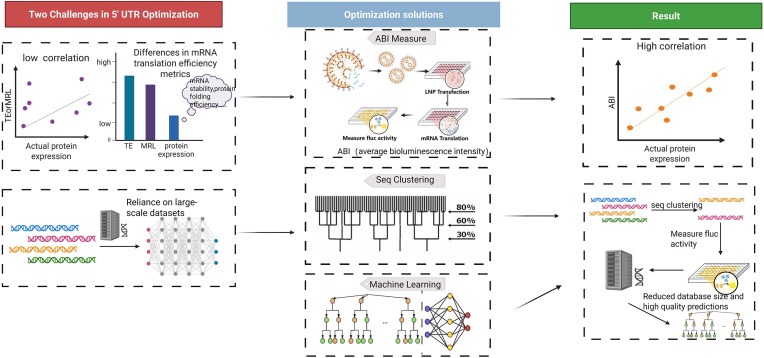

## Introduction

Protein synthesis involves two major steps: transcription of DNA into messenger RNA (mRNA) by RNA polymerase, followed by translation of mRNA into functional proteins via ribosome-mediated assembly of amino acids [1]. The 5′ untranslated region (5′UTR), a critical regulatory element upstream of the coding sequence, plays a central role in modulating translation efficiency (TE) by controlling ribosome recruitment and initiation. It contains *cis-*acting elements such as upstream open reading frames and internal ribosome entry sites, while its secondary structure can either enhance or inhibit ribosome binding, directly impacting protein expression levels [2]. In mRNA therapeutics and vaccines, 5′UTR optimization has become indispensable for maximizing protein production. For example, engineered 5′UTRs in COVID-19 mRNA vaccines (e.g. Pfizer-BioNTech and Moderna) significantly improved antigen expression to elicit robust immune responses [3]. Similarly, in gene therapy, tailored 5′UTRs enhance transgene expression and therapeutic protein yields, underscoring their pivotal role in advancing mRNA-based biomedical applications [4].

The function of *cis-*elements within 5′UTR has been individually characterized [2]. However, unexpected interactions among these elements may reduce the TE of a well-designed 5′UTR [2, 5, 6]. As a result, manually altering *cis-*elements in the 5′UTR may not yield the anticipated level of effectiveness [2, 5, 6]. Conversely, machine learning approaches can reveal the hidden connections among *cis-*elements, thus aiding in precise predictions of the efficiency of 5′UTR [7]. Consequently, multiple 5′UTR libraries have been constructed to train machine learning models [7–11]. Nevertheless, these models encounter two conflicting challenges.

On the one hand, the training datasets have not succeeded in establishing a direct link between translational efficiency and the actual protein expression level. Currently, mean ribosome load (MRL) and TE serve as the primary metrics in these datasets to assess the effects of 5′UTRs on translation [7–9, 11]. MRL represents the average ribosome count attached to an mRNA, while TE is estimated as the ratio of ribosome-protected mRNA fragments to total mRNA molecules determined by RNA sequencing [12]. However, these metrics are limited in scope; they only reflect the ribosome binding stage of protein synthesis and fail to capture the final protein output. Additionally, high MRL can lead to reduced mRNA stability, resulting in lower total protein output due to a short half-life [13]. The significance of TE lies in its ability to reveal the efficiency of mRNA translation into proteins at a specific moment, but it is merely an instantaneous measurement with low reproducibility [12]. Consequently, neither metric accurately portrays the real protein levels. Furthermore, the recombinase-mediated high-throughput approach integrates DNA fragments into the chromosome, allowing the expressed protein to encompass both transcription and translation intensities. Thus, it does not truly measure translational efficiency either [8]. Hence, it is crucial to develop a high-throughput method for quantifying the TE of each 5′UTR.

On the other hand, a precise method for quantifying 5′UTR necessitates significantly costlier libraries. For instance, in the case of mRNA vaccines, the DNA fragment is transcribed *in vitro*, and the resulting mRNA is then delivered into the human body for *in vivo* translation [3]. However, an effective approach that replicates this process necessitates separate measurement of each 5′UTR in individual microtubes, leading to significantly higher costs.

**Figure 1.**
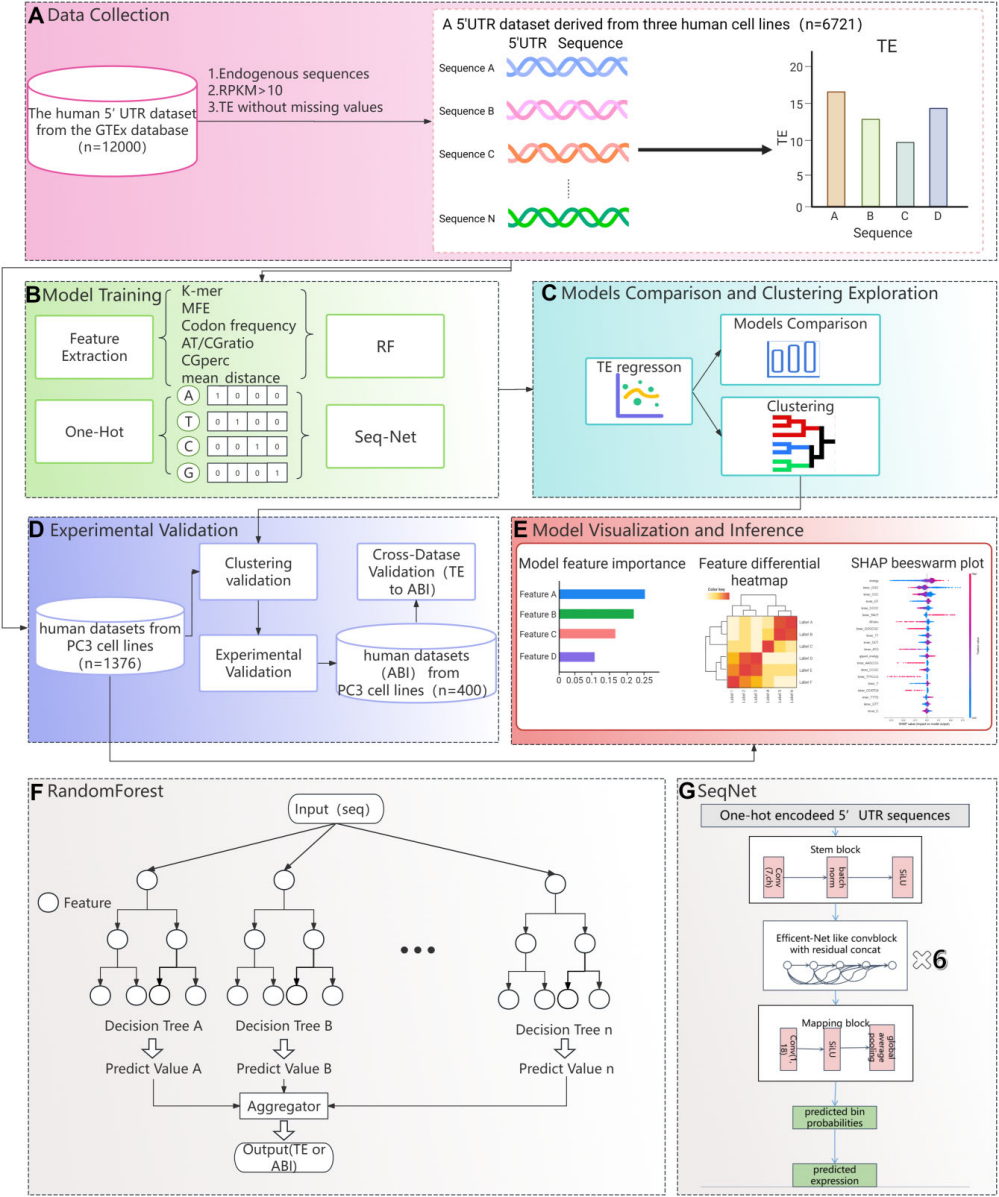
Research flowchart. (**A**) Data collection. A dataset (*n* = 6721) was selected from the GTEx project database analyzed by Cao *et al.* (*n* = 12 000) [8]. (**B**) Model training. Sequence information was processed through feature extraction to obtain biological characteristics and one-hot encoding, which served as inputs for the RF model and SeqNet model. (**C**) Model comparison and clustering exploration. Trained models were compared with the baseline RF-Cao model [8], and clustering schemes were investigated. (**D**) Wet-lab validation. PC3 cell line data (*n* = 1376) from the filtered dataset (*n* = 6721) were used to validate clustering schemes. Average bioluminescence intensity (ABI) measurements were performed for cross-dataset validation, comparing the predictive performance between models trained on ABI datasets and TE-value datasets for protein expression levels. (**E**) Model visualization and interpretive analysis. Native importance maps, gene differential heatmaps, and SHAP plots were employed to investigate patterns learned by the models. (**F**) RF model diagram. (**G**) SeqNet framework diagram. Panels (**A**) and (**E**) were created by BioRender. LINFE, W. (2025) https://BioRender.com/1ug6hzm and https://biorender.com/744eikh.

In this study, we addressed the challenges of predicting 5′UTR-mediated TE by developing computational and experimental approaches (Fig. 1 and Supplementary Table S1). To overcome the limitations of manual 5′UTR engineering, two models from 6721 5′UTR sequences were constructed: a random forest (RF) model incorporating both sequence and RNA secondary structure features and a novel SeqNet model that leverages sequence features to predict TE. Recognizing the resource-intensive nature of training these models on large datasets, we further developed an innovative sequence clustering strategy that significantly reduces the required training dataset size while maintaining predictive accuracy. Finally, a small library based on firefly luciferase expression was used to verify that this method could more accurately reflect real-world protein expression levels in mRNA therapeutics than the TE value. This method closely mimics the functional process of mRNA vaccines, enabling precise quantification of protein output. The effectiveness of our integrated approach was validated through proof-of-concept experiments in PC3 cell lines (Fig. 1D), which demonstrated both the predictive power of our models and the reliability of our experimental system. We believe this strategy, combining advanced machine learning techniques with high-fidelity experimental validation, not only could advance the field of 5′UTR optimization but also provide a framework that could be adapted to other areas of molecular biology.

## Materials and methods

### Dataset for model training

The datasets used for each step are presented in Supplementary Table S1. Three endogenous human 5′UTR datasets derived from HEK293T, PC3, and muscle cell lines, curated by Cao *et al.* [8], were obtained to train the model. Among these datasets, 6721 5′UTR sequences were selected based on the following criteria: (i) mRNA sequences with missing TE values were removed (Fig. 1A). (ii) Only mRNA sequences with RPKM (reads per kilobase of transcript per million mapped reads) exceeding 10 were included. This cutoff was determined based on prior studies [14], which established that transcripts with RPKM ≤10 exhibit elevated noise sensitivity in TE calculations. Such low-abundance mRNAs were excluded because their shallow sequencing depth amplifies two critical sources of variability: (i) low read counts subject TE values (Ribo-seq/RNA-seq ratios) to Poisson noise dominance, where minor count fluctuations lead to drastic TE value variations; (ii) low read counts are highly susceptible to experimental biases (e.g. partial RNA degradation), causing TE values to deviate from true translation states. Consequently, TE values derived from these sequences would introduce erroneous labels during the training process, thereby disrupting the model’s ability to learn authentic TE regulatory patterns.

### Overview of the RF model

The RF regression model was implemented in Python 3.8.18 using scikit-learn (v1.3.0), comprising 100 decision trees (n_estimators = 100) with key hyperparameters including max_features = 0.8 and min_samples_leaf = 1.

To optimize these hyperparameters, a grid search was performed with the following candidate parameter ranges:

n_estimators: [50, 100, 150]

min_samples_leaf: [1, 2, 4]

max_features: [0.5, 0.8]

Other parameters, such as max_depth and min_samples_split, were kept at default values (max_depth = None, min_samples_split = 2) based on preliminary experiments. The optimal combination was selected via 10-fold cross-validation using mean squared error (MSE) as the evaluation metric. The final model parameters (n_estimators = 100, max_features = 0.8, min_samples_leaf = 1) achieved the lowest MSE, balancing model complexity and generalization performance.

Feature space construction utilized Biopython (v1.78) to extract the following parameters: codon usage frequencies, *k*-mer frequencies (*k* = 1–6), upstream start codon counts upstream of main start codons, and GC content metrics. Structural descriptors were supplemented using ViennaRNA (v2.6.2), including minimum free energy (MFE), G-quadruplex ΔMFE values, and base-pairing distances (Fig. 1B).

To control the randomness of each decision tree, the ExtraTreeRegressor was employed [15]. The final predictions were derived from averaging the outputs of all decision trees. This ensemble learning approach effectively leverages the independence of multiple trees, reducing the risk of overfitting while enhancing the model's ability to fit complex data patterns (Fig. 1F).

### Overview of the SeqNet model

To address challenges in modeling TE and gene expression, the LegNet framework [16] was enhanced through two key modifications to generate the SeqNet model. First, the original Adam optimizer was replaced with the Lion optimizer [17] to improve training stability and convergence, enabling better capture of subtle regulatory signals in 5′UTR sequences despite inherent gradient noise in TE data. Second, a one-hot encoding scheme with dynamic padding (rather than truncation) was implemented to accommodate variable-length 5′UTR sequences (≤200 bp), maintaining sequence integrity while ensuring consistent input dimensions (Fig. 1B). These combined improvements in optimization strategy and sequence encoding established the computational foundation for the SeqNet model.

The SeqNet soft classification model represents an innovative architectural approach that reformulates the regression problem of gene sequence-to-expression prediction as a probabilistic classification task [16]. Instead of directly predicting absolute expression values, the model generates a probability vector indicating the likelihood of a sequence belonging to each predefined expression bin. SeqNet adopts a probabilistic framework where the experimentally measured expression value (denoted as *e*, representing the mean expression bin value) is assumed to follow a normal distribution with *μ* = *e* + 0.5 and *σ* = 0.5.


(1) \begin{eqnarray*}
\rho \sim N\left( {\mu = e + 0.5,{\mathrm{\ }}{\rm sd} = 0.5} \right).
\end{eqnarray*}

For each defined category (*i*) (corresponding to an expression level bin), the probability of the category is determined by the cumulative probability within the interval [*i*, *i* + 1). Specifically, the intervals for category 0 and category 28 are defined as (-∞, 1] and [28, +∞), respectively. To obtain the predicted expression value, the model multiplies these probabilities by their corresponding bin numbers. The expression value is defined as:


(2) \begin{eqnarray*}
{\rm Expression} = \mathop \sum \limits_{i = 1}^n \left( {{\rm probs}\,{\mathrm{*}}\,{\rm bins}} \right).
\end{eqnarray*}

### SeqNet architecture

The SeqNet architecture was developed as a fully convolutional neural network derived from EfficientNetV2, with selected features incorporated from DenseNet and additional custom modules. The first module (stem block) was constructed with a standard convolution layer, followed by batch normalization (BatchNorm) and the SiLU activation function. A sequence of EfficientNet-like convolutional modules was then implemented, where group convolution was employed to replace the depthwise convolution used in the original EfficientNetV2. The architecture was completed with a final module containing a pointwise convolution layer, followed by global average pooling and a SoftMax activation function (Fig. 1G).

The first SeqNet block (stem block) is a standard convolution with a kernel size of 7. The output is passed through a series of six convolutional blocks, where standard residual connections are replaced with channel-wise residual connections. All convolutions use the “same” padding mode. The resizing blocks have the same structure as the Stem block. The squeeze-and-excitation (SE) blocks in the EfficientNet-like blocks are modified versions of those in EfficientNetV2. Specifically, the bilinear block within the SE blocks is compressed using a low-rank representation derived from canonical polyadic decomposition, implemented via the TensorLy library. This decomposition reduces the number of trainable parameters while preserving feature recalibration capabilities. The final block consists of a single pointwise convolution layer, followed by channel-wise global average pooling and a SoftMax activation function. We used 256 channels for the first block and [128, 128, 64, 64, 64, 64] channels for the six EfficientNetV2-like blocks, respectively. During training, the Kullback–Leibler divergence between the distribution derived from the training data and the model’s output vector is used as the model loss to predict the probability of each category (bin) (Supplementary Fig. S2).

The SeqNet model adopts a soft classification framework for key advantages over regression models: (i) probabilistic uncertainty: the soft classification approach inherently captures uncertainty through probability distributions, making it more robust to noisy biological data; (ii) loss alignment: cross-entropy better matches discrete gene expression; (iii) implicit regularization: the categorical output space introduces natural boundaries, preventing overfitting to extreme outliers common in high-throughput experiments; and (iv) adaptive discretization: empirical analysis showed that discretizing continuous expression values into probabilistic classes improved generalization on sparse/noisy datasets while retaining the capacity to resolve fine-grained differences through the neural network’s feature learning. This architecture thus achieves superior stability and accuracy by leveraging deep learning’s representational power within a probabilistically grounded framework.

### Clustering analysis

Deep learning has emerged as a promising approach for constructing sequence-to-expression models. By learning patterns and associations from large datasets, these models can predict or explain the expression levels of new data. However, the model’s dependency on large-scale datasets poses significant cost barriers, which creates a substantial challenge for laboratories with limited resources.

To address this issue, a clustering-based strategy was implemented. The approach was grounded in an intuitive hypothesis: When two mRNAs exhibit sequence similarity, their shared structural and functional features may lead to comparable expression levels. Sequence similarity analysis was performed through clustering, which identified and removed highly redundant data while ensuring the selected subset maximally preserved the diversity and critical regulatory patterns of the original dataset.

The resulting representative dataset, though smaller in size, demonstrated higher information density and broader coverage. This optimized dataset enabled models to learn core patterns more efficiently. By improving data quality (enhancing diversity and reducing redundancy), clustering allowed models to achieve better performance with relatively small datasets, thereby decreasing their dependence on large-scale data. Measurement of this representative subset for the costly new metric (ABI) facilitated the construction of a new database for model training.

Based on this hypothesis, CD-HIT was employed to perform clustering analysis on data filtered from Cao *et al.* [8]. CD-HIT utilizes a greedy incremental clustering method: input sequences are first sorted by length and processed from longest to shortest. The longest sequence automatically becomes the representative sequence of the first cluster. Each subsequent sequence is then compared with existing representative sequences and assigned to an appropriate cluster or designated as the representative of a new cluster based on sequence similarity. This iterative process continues until all sequences are clustered.

To evaluate clustering quality (Fig. 1C), a specific test set was designed to maximize sequence diversity to assess clustering performance. By adjusting the clustering schemes, training sets of varying sizes were constructed (Supplementary Table S1). The model’s predictive performance was monitored as the training set size increased, aiming to identify the optimal balance between model compression rate and accuracy restoration.

### Model evaluation

To evaluate the models (Fig. 1C), both standard 10-fold cross-validation and random 10-fold cross-validation were employed (Supplementary Table S1). In standard 10-fold cross-validation, the dataset was divided into 10 fixed, non-overlapping subsets, with each subset serving as the test set once while the remaining nine subsets were used for training. This process was repeated 10 times, ensuring that each data point participated in both training and validation [17]. In contrast, random 10-fold cross-validation introduced variability by shuffling the data prior to each partitioning, resulting in different subsets for each iteration.

The Pearson correlation coefficient was used to measure the model’s performance. The Pearson correlation coefficient (*r*) is an indicator of the strength of the linear relationship between predicted values and actual observations. Its calculation formula is as follows:


(3) \begin{eqnarray*}
r = \frac{{\mathop \sum \nolimits_{i = 1}^n \left( {{{X}_i} - \bar{X}} \right)\left( {{{Y}_i} - \bar{Y}} \right)}}{{\sqrt {\mathop \sum \nolimits_{i = 1}^n {{{\left( {{{X}_i} - \bar{X}} \right)}}^2}} \sqrt {\mathop \sum \nolimits_{i = 1}^n {{{\left( {{{Y}_i} - \bar{Y}} \right)}}^2}} }}.
\end{eqnarray*}

Here, *X_i_* represents the predicted values from the model, and *Y_i_* represents the actual observed values. $\bar{X}$ and $\bar{Y}$ denote the mean values of the predicted and observed values, respectively. When *r* is close to 1, it indicates a strong positive linear relationship between the predicted and observed values; when *r* is close to -1, it indicates a strong negative linear relationship; and when *r* is close to 0, it suggests no linear relationship between the two.

### Construction of the 5′UTR plasmid library

The 5′UTRs were polymerase chain reaction (PCR) amplified, digested, and ligated into the plasmid containing a firefly luciferase reporter gene. The ligated plasmids were transformed into DH5α competent cells via chemical transformation. The transformed cells were plated on selective media containing kanamycin for screening. Positive clones were verified by colony PCR and sequencing to confirm the successful insertion of the 5′UTR gene sequences. The correctly sequenced colonies were expanded, and plasmids were extracted (Vazyme Plasmid Mini Kit, DC221-01), ultimately yielding the plasmid library. Plasmid containing the reference 5′UTR sequence was synthesized by GenScript Biotech Corporation (GenScript, C5659GK200).

### Preparation of firefly luciferase mRNA transcripts

All mRNAs used in this study contained a cap-1 structure m7G(5′)pppA(2′-O-Me)pG (Hongene Biotech, ON-134) and a 120-nt poly(A) tail. The DNA templates of mRNAs were amplified by PCR using a forward primer and a reverse primer containing 120 thymidine residues at the 5′ end (Supplementary Table S2). The DNA templates were purified using the Mag-MK 96 Well PCR Products Purification Kit (Sangon Biotech, B518753) and examined by agarose gel electrophoresis. mRNAs were synthesized by *in vitro* transcription with complete substitution of UTP by *N*1-methyl-pseudouridine-5′-triphosphate (Hongene Biotech, R5-027) using the High Yield T7 RNA Synthesis Kit (Hongene Biotech, ON-040) according to the manufacturer’s instructions. Briefly, a 10 μl transcription reaction contained a maximum of 0.5 μg DNA template, 8 mM each of ATP, CTP, GTP, and N1-methyl-pseudouridine-5′-triphosphate, m7G(5′)pppA(2′-O-Me)pG cap analog solution, 100 U T7 Enzyme Mix, and 1× T7 Reaction Buffer. After 2-h incubation at 37°C, the DNA template was digested by adding 1 U DNase I for 15 min at the same temperature. The mRNA products were purified using Dynabeads MyOne™ Carboxylic Acid beads (Thermo Fisher Scientific, 65011). The concentration of purified mRNA was measured using a NanoDrop 2000 spectrophotometer (Thermo Fisher Scientific), with recording of A_260_/A_280_ and A_260_/A_230_ ratios. All mRNAs were diluted to working concentrations in 50 mM citrate buffer (pH 4.0) and stored at −80°C until further use.

The integrity and size distribution of mRNA were analyzed using capillary electrophoresis (Bioptic Qsep100) with an RNA analysis cartridge (Bioptic, R1). Residual T7 RNA polymerase and double-stranded RNA (dsRNA) contaminants were detected using the T7 RNA Polymerase Residual Detection ELISA Kit (Hzymes, HBP006004) and the dsRNA Quantitative Detection ELISA Kit (Hzymes, HBP003802), respectively.

### Synthesis of lipid nanoparticles

Lipid nanoparticles (LNPs) were prepared by rapid mixing of the ethanol and aqueous phases at a 1:3 (v/v) ratio via vortex mixing. The ethanol phase contained the ionizable lipid SM102, DSPC, cholesterol, and DMG-PEG2000 at a molar ratio of 50:10:38.5:1.5. The aqueous phase was prepared by dissolving synthesized firefly luciferase mRNA (containing the target 5′UTR sequence, see prior section) in 50 mM citrate buffer (pH 4.0). The mRNA encapsulation efficiency in LNPs was quantified using the Quant-iT™ RiboGreen™ RNA Assay Kit (Thermo Fisher Scientific, R11490) according to the manufacturer’s protocol. The hydrodynamic diameter and polydispersity index of LNPs were determined by dynamic light scattering at 25°C in 50 mM citrate buffer (pH 4.0) using a dynamic light scattering instrument (Anton Paar, Litesizer 100).

### Firefly luciferase assay *in vitro*

HeLa cells were cultured in 37°C incubators supplemented with 5% CO_2_. For the mRNA transfection, 1.5 × 10^4^ HeLa cells were seeded per well in a 96-well plate. The cells were maintained in 100 μl of Dulbecco’s modified Eagle medium (DMEM, Gibco) supplemented with 10% fetal bovine serum (FBS, Gibco) and 1% penicillin–streptomycin (Gibco). The cells were incubated at 37°C for 24 h. A total of 100 ng of the aforementioned mRNA–LNP complexes were dispersed in 1× phosphate buffered saline and added to the cell-containing wells. Three biological replicates were included for each mRNA formulation. Following a 24-h incubation at 37°C, the 96-well plate was allowed to reach room temperature for a period of 30 min. Subsequently, 100 μl of Firefly Glo Luciferase Assay Substrate (Yeasen, 11404ES) was added to each well, followed by gentle shaking in the dark for 5–10 min. The supernatant of each well was then transferred to a white 96-well plate (Thermo Fisher Scientific, 436 110), and bioluminescence was quantified using a multimode microplate reader (Tecan, Infinite200-PRO_E_Plex).

### Data processing and bioluminescence normalization

For each 5′UTR sample, three independent replicate plates were prepared, with each plate containing a reference well. Bioluminescence signals were measured in duplicate and the average value taken. To compensate for variations in cell density and health status, firefly luciferase expression was normalized. The ABI for each well was calculated by dividing its actual firefly luciferase expression level by the expression level of the reference well in the same 96-well plate. The final ABI value represents the average of this normalized ratio across the three independent replicate plates.


(4) \begin{eqnarray*}
{\rm ABI} = \frac{1}{n}\mathop \sum \limits_{i = 1}^n \frac{{{\rm experimental}\_{\rm BI}_i}}{{{\rm reference}\_{\rm BI}_i}},\quad n = 3.\
\end{eqnarray*}

## Results

### Establishment of the RF model and SeqNet model

**Figure 2.**
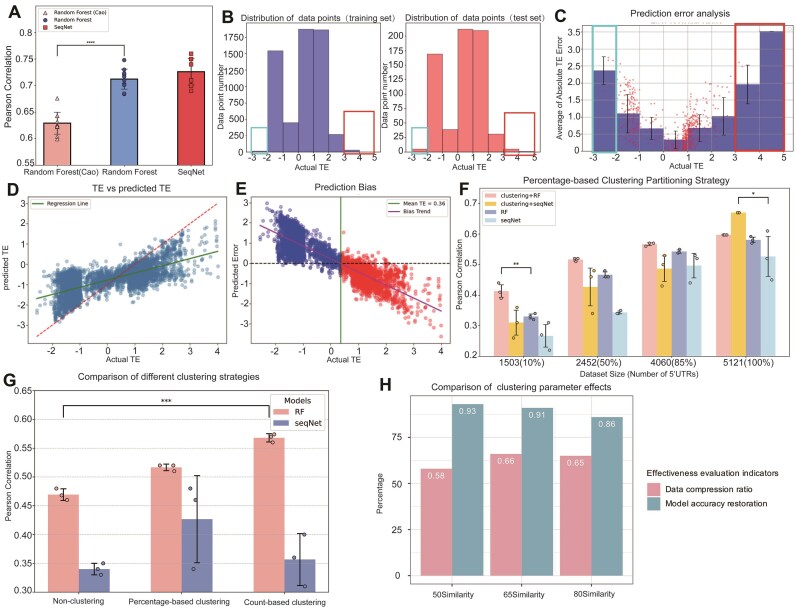
(**A**) Three models were compared for performance evaluation. The performance of baseline RF-Cao model (pink), RF model (blue), and the SeqNet model (red) was compared by 10-fold cross-validation. (**B**) Distribution of the data points. The distribution of training set is displayed in the left panel, and test set is in the right panel. The rectangles indicate the TE value intervals where data points are relatively scarce. (**C**) Average of the Absolute TE Error. The bar height represents the average of absolute difference between predicted and measured TE values, with negative TE values resulting from logarithmic transformation. The rectangles indicate the TE value intervals where data points are relatively scarce. (**D**) Predicted TE values (*y*-axis) against actual TE values (*x*-axis), with red and green lines indicating standard and fitted lines, respectively. (**E**) Predicted error (*y*-axis) versus actual TE values (*x*-axis), featuring median TE demarcation (green line) and regression fit (purple line). (**F**) Prediction accuracy is contrasted between non-clustered and percentage-clustered training sets, with *x*-axis showing dataset size and *y*-axis Pearson correlation coefficients (pink/yellow bars for clustered models, dark/light blue for unclustered). (**G**) Three clustering strategies are compared: non-clustering group, percentage-based clustering group, and count-based clustering group, evaluated using both RF and SeqNet models. (**H**) Clustering parameter effects are analyzed, with *x*-axis representing experimental groups at varying similarity thresholds and *y*-axis showing compression rates versus accuracy restoration. *P*-values were derived from one-way ANOVA, with ****P ≤ .0001, ***P ≤ .001, **P ≤ .01, and *P ≤ .05.

The dataset used in this section was comprised of a total of 6721 5′UTR sequences annotated with TE values [8, 9]. Sequence features were extracted from these 5′UTRs, including *k*-mer frequency, AT ratio, CG ratio, CG content, and the minimum free energy of secondary structures. These features served as the basis for RF decision tree construction [18]. Model performance was evaluated based on the average Pearson correlation coefficient across the 10 folds. As shown in Fig. 2A, our RF model achieved a Pearson correlation of 0.712, demonstrating significant improvement over the previously reported RF model [8].

Next, we adapted the LegNet model by modifying its downstream prediction task to predict TE values and further improved the loss function, resulting in the design of the SeqNet model [16]. The SeqNet model achieved a Pearson correlation coefficient of 0.726 on the same dataset (Fig. 2A). On the large dataset of 6721 sequences, the SeqNet model demonstrated superior performance compared to the RF model. This is because the CNN(Convolutional Neural Networks)-based architecture of SeqNet can better leverage large-scale data to fit complex parameters, thereby exhibiting stronger fitting capabilities.

### Exploration of pattern clustering in library construction

Constructing a 5′UTR dataset for training machine learning models requires a substantial amount of data. However, it is widely acknowledged that the quality of training datasets is directly correlated with the costs associated with their development. Therefore, our objective is to explore methods to reduce the model’s dependency on large-scale data before developing a high-quality library that can accurately illustrate the 5′UTR’s impact on translation.

During model establishment, both standard 10-fold cross-validation and randomized 10-fold cross-validation were implemented for performance evaluation. The randomized approach consistently demonstrated superior results, which subsequent analysis attributed to the dataset’s data point distribution. The enhanced randomness in randomized 10-fold cross-validation promotes greater similarity in TE value distributions between training and test sets (Fig. 2B). Figure 2B and C demonstrate the distribution patterns of both training and test sets, along with the mean absolute errors across different TE value ranges. The following observations can be made: (i) In the TE value ranges of (-3, -2) and [3, 5], the training set contains limited data points, which leads to insufficient training resources and consequently higher errors in the test set; (ii) for the TE value range of (-2, 3), the training set provides adequate coverage, resulting in lower errors in the test set.

The observed errors at the extremes of the TE spectrum likely stem from two primary factors. First, the training set contains insufficient representation of extreme values (particularly for TE < -2 and TE > 3), which limits the model’s ability to learn these boundary regions effectively. Second, machine learning models exhibit an inherent systematic regression bias—as documented in recent literature [19]—where predictions tend to regress toward the mean to optimize Pearson’s correlation coefficient. As demonstrated in Fig. 2D, the green fitted line displays a shallower slope compared to the standard reference line, indicating that the model systematically overestimates low TE values while underestimating high TE values. This observation receives further support from the prediction bias analysis in Fig. 2E: For low TE values, the prediction error (predicted minus actual) remains consistently positive, confirming the overestimation tendency; conversely, for high TE values, the error becomes negative, demonstrating systematic underestimation. Notably, this bias pattern persists even in regions with relatively adequate training data coverage (e.g. TE ∈ (-2, -1)). These findings underscore that high prediction accuracy and robust generalization capabilities require not only sophisticated model architecture but also a training set with comprehensive and balanced representation across the entire value spectrum.

### Clustering reduces dataset redundancy and compresses dataset size

From a machine learning perspective, our analytical results demonstrated a strong correlation between TE value and sequence similarity. Specifically, sequences with high similarity tend to cluster within identical TE value intervals. This observation aligns with the biological principle that as sequence similarity increases, the variations in their regulatory elements decrease, leading to more uniform regulatory mechanisms [1]. The identified correlation enables strategic downsizing of training sets through the exclusion of highly similar 5′UTR sequences while maintaining model predictive accuracy. Consequently, the optimal dataset for such analyses should exhibit both uniform distribution characteristics and abroad sequence diversity spectrum.

We implemented clustering to reduce the training set size while tracking model accuracy variations and preserving sequence diversity. CD-HIT [20] was applied, with a 50% similarity threshold, to cluster 6721 5′UTR sequences into 1600 distinct groups. To select representative sequences from each cluster, CD-HIT employs a length-based prioritization strategy. The algorithm first sorts all input sequences in descending order of length, ensuring longer sequences have priority for selection as cluster representatives. Each subsequent sequence is then evaluated sequentially: if it demonstrates sufficient similarity (exceeding the predefined threshold) to any existing cluster representative, it is assigned to that cluster. When no suitable match is found, the current sequence establishes a new cluster as its representative. Following these selection criteria, we constructed a test set comprising 883 representative sequences from each cluster to evaluate clustering performance. The selection process specifically excluded singleton clusters (those containing only one sequence) since these sequences lacked corresponding training data for effective model learning. This approach ensured both adequate sequence diversity and optimal conditions for model training.

Subsequently, we evaluated two clustering strategies: percentage-based clustering and count-based clustering. In the percentage-based approach, 5′UTRs were randomly selected from each cluster at fixed proportions (10%, 50%, or 100%) to generate training sets of varying sizes. (Fig. 2F; see Supplementary Fig. S3 for the size of other datasets). Our analysis revealed three key findings: First, the clustering + RF group (RF model trained on clustered data) demonstrated significantly better performance than the RF group (RF trained on non-clustered data of equivalent size), with this advantage being particularly pronounced in smaller datasets. Second, RF outperformed SeqNet in smaller datasets, and RF + clustering showed superior results compared to SeqNet + clustering (SeqNet trained on clustered data). Third, while SeqNet’s performance improved substantially with larger datasets, the clustering + RF group exhibited more gradual improvement. Notably, the clustering + SeqNet group achieved optimal performance across all dataset sizes. These results confirm that clustering effectively reduces dataset redundancy while maintaining model performance.

An extreme scenario occurred in percentage-based clustering where some clusters contained thousands of sequences. In such cases, selecting a percentage of data could easily result in redundant data from large clusters. To address this issue, count-based clustering was implemented, where six 5′UTR sequences were selected from each cluster. When clusters contained fewer than six sequences, all available sequences were included. This approach generated a training set of 2468 sequences. Comparative analysis revealed that count-based clustering demonstrated significant performance improvement over both percentage-based clustering and the non-clustered approach (Fig. 2G).

In addition to the above analysis, we examined clustering effects across varying similarity thresholds, identifying solutions with data compression rates exceeding 50% and selecting the method that maximized model accuracy restoration. As illustrated in Fig. 2H, a 50% similarity threshold produced optimal results. This method achieved a 58% data compression rate (reducing the dataset from 5838 to 2468 sequences) while preserving 93% of the model’s accuracy relative to the full dataset. Although some small clusters may individually lack sufficient representation, the collective contribution of these clusters, through retention of all their sequences, ensured comprehensive coverage of the sequence space. Overall, our strategy preserved the full regulatory landscape of the dataset while compressing its size by approximately one-third, demonstrating that even sparsely sampled clusters collectively contribute to global functional representation.

### Necessity of constructing an average bioluminescence intensity-based library

**Figure 3.**
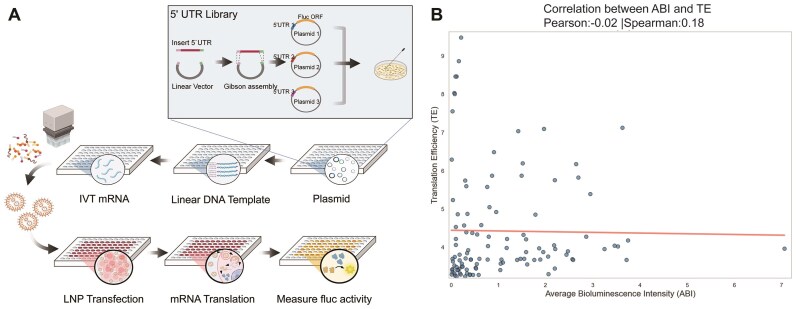
Schematic diagram of the 5′UTR library construction strategy. (**A**) Experimental workflow. 5′UTRs were amplified by PCR and ligated into the expression plasmids. The resulting plasmids were linearized, polyadenylated, co-transcriptionally capped, and transfected into HeLa cells. The mRNA TE was measured by bioluminescence of expressed firefly luciferase. (**B**) Correlation between ABI and TE. The *x*-axis represents the fluorescence intensity measured in wet-lab experiments (normalized), and the *y*-axis represents the annotated TE values from the database.

Intracellular protein expression may vary when the same gene is expressed from plasmids, genomic DNA, or mRNA. To accurately measure translation intensity from mRNA, we employed a firefly luciferase-based bioluminescence system that replicates the functional mechanism of mRNA vaccines (Fig. 3A). Specifically, each 5′UTR was inserted into a plasmid upstream of a firefly luciferase reporter gene. Using an automated liquid handling system, plasmids underwent sequential linearization, polyadenylation, co-transcriptional capping *in vitro*, and encapsulation into LNPs (Supplementary Fig. S1). Pre-experimental quality control of automated system outputs included RNA analysis (integrity, % dsRNA, % T7 enzyme residual) and LNP characterization (particle size, encapsulation efficiency) (Supplementary Fig. S4). The analysis verified that all parameters remained within predefined quality thresholds, confirming the system’s consistent compliance with required standards. These findings validate the reproducibility and reliability of both the *in vitro* transcription system and LNP encapsulation process.

While acknowledging potential cell-type-specific effects on translation, HeLa cells were selected for primary screening and model training based on critical operational and biological considerations. HeLa cells provide superior adherence and consistent growth essential for high-throughput LNP transfection in 96-well formats using automated platforms, whereas alternatives like HEK293T exhibit vulnerability to cell loss during processing [8, 10, 21]. Furthermore, HeLa represents a well-established model for mRNA-LNP delivery studies, with extensive literature documenting its reliable and efficient mRNA expression under LNP transfection conditions [22–26]. Preliminary validation using representative 5′UTRs showed consistent expression trends between HeLa and HEK293T cells (Supplementary Fig. S5), supporting HeLa’s suitability for this screening platform.

The encapsulated mRNA was transfected into a well of a 96-well plate containing HeLa cells. Bioluminescence was quantified using a multimode microplate reader at 24 h post-transfection, the optimal timepoint determined by pre-experimental multi-timepoint assessment (13–30 h), based on firefly luciferase kinetics and literature consensus [27–30], which showed no statistically significant difference between 24 and 30 h (Supplementary Fig. S6). To account for inter-well variability in firefly luciferase expression, all raw bioluminescence signals were normalized to the corresponding reference well on the same plate. The normalized ABI was then used for downstream comparative analysis across samples, as outlined in Equation 4 in the “Materials and methods” section. Based on this method, a library consisting of 110 5′UTR sequences was constructed. The protein expression level was characterized by the ABI intensity. As expected, an exceptionally weak correlation between TE values and ABI was observed, indicating the TE values are not a suitable metric to capture the TE (Fig. 3B). To validate the reliability of the low correlation between ABI and TE values, we performed a power analysis (Supplementary Fig. S7). With 400 sequences, the observed power reached 0.995, confirming the authenticity and robustness of this finding.

### Validation of clustering strategy

**Figure 4.**
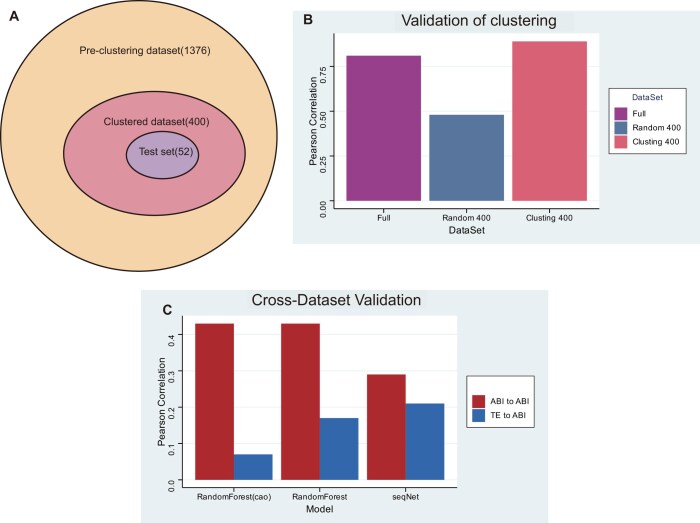
Validation of clustering strategy and wet-lab experiments in the PC3 cell line. (**A**) Schematic diagram of clustering in the PC3 cell line. (**B**) Validation of clustering performance in the PC3 cell line. (**C**) Performance comparison of cross-dataset TE and ABI prediction models.

The clustering approach was validated using a subset of 1376 5′UTR sequences, which included all the PC3 cell line 5′UTR sequences. We conducted sequence similarity clustering on these 1376 PC3-specific 5′UTR sequences and curated a clustered dataset of 400 sequences, with 348 allocated for training and 52 for testing (Fig. 4A). Notably, when utilizing the entire set of 1376 5′UTR sequences to predict the test set, the Pearson correlation coefficient was 0.76 (Fig. 4B). In contrast, a randomly chosen dataset of 348 5′UTR sequences yielded a correlation of only 0.48, while the clustered dataset of 348 5′UTR sequences achieved a Pearson correlation coefficient of 0.89 when predicting the test set (Fig. 4B).

Following the computational validation of the clustering of 5′UTRs from PC3 cell lines, an experimental validation was also performed. A library composed of the clustered 400 5′UTR sequences was constructed, and the ABI was measured (Fig. 4C). The results showed that when predicting ABI, the RF model trained on TE values achieved a Pearson correlation coefficient of 0.17, while the model trained on ABI achieved a correlation of 0.43. Similarly, the Pearson correlation coefficient for RF-Cao improved from 0.07 to 0.43. This further demonstrates that the TE value dataset is not an optimal dataset for models to identify sequences with high protein expression levels.

## Discussion

In this study, we developed two complementary models: an RF model suitable for small datasets and a CNN-based SeqNet designed for large-scale data. To enhance the efficiency of our models, we implemented sequence clustering to reduce dataset redundancy. We validated our approach using a firefly luciferase-based bioluminescence reporter library, which simulated the mRNA vaccine workflow to optimize 5′UTR sequences for improved protein expression. To achieve this, we ligated the 5′UTRs to a firefly luciferase reporter gene. This was followed by *in vitro* transcription, co-transcriptional capping, LNP encapsulation, and transfection into HeLa cells, enabling high-throughput bioluminescence quantification. These experimental strategies are crucial for optimizing mRNA, as the ability to directly measure protein expression levels is essential for enhancing both TE and the therapeutic potential of mRNA-based therapies.

There are two critical challenges in 5′UTR optimization: the limitations of traditional TE metrics (TE/MRL) and the high cost of large-scale library construction. Traditional metrics like TE and MRL often fail to accurately reflect protein expression levels, as evidenced by the weak correlation between TE values and ABI (Fig. 3B). The weak correlation between TE and ABI likely arises because TE reflects translation initiation/elongation efficiency, while ABI integrates additional regulatory layers such as mRNA stability, transcript half-life, and post-translational processes that collectively govern protein abundance. This underscores the necessity for direct protein expression measurements, such as bioluminescence, in evaluating 5′UTRs. Moreover, the introduction of clustering significantly reduced the dataset size while maintaining model accuracy. The clustered dataset encompasses sufficient sequence diversity with a smaller training set to enable models to learn key regulatory patterns, thereby critically reducing the experimental burden and cost associated with measuring novel metrics like ABI across the entire dataset. By focusing measurements on this compact yet representative subset, we avoided redundant experiments while preserving biologically meaningful variation.

Although SeqNet’s performance appeared suboptimal at smaller dataset sizes compared to the RF model, this did not imply that clustering was ineffective on this model. On the contrary, SeqNet was underfitted on small datasets. As the dataset size increased, the effect of clustering on SeqNet became increasingly significant, eventually surpassing the clustering + RF group. Overall, we believe the dataset should contain as diverse a set of sequences as possible in order to establish a concise yet high-quality library. Additionally, the higher the similarity threshold used for clustering, the more clusters will be generated, and the larger the dataset size will become. However, this also leads to higher model accuracy. In terms of efficiency in reducing dataset size, count-based clustering outperforms percentage-based clustering and non-clustering (Fig. 2G), ultimately compressing the dataset to one-third of its original size compared to non-clustering without affecting model accuracy. Together, these strategies provide an expandable framework for optimizing 5′UTRs, advancing mRNA therapies toward greater precision and practical utility.

**Figure 5.**
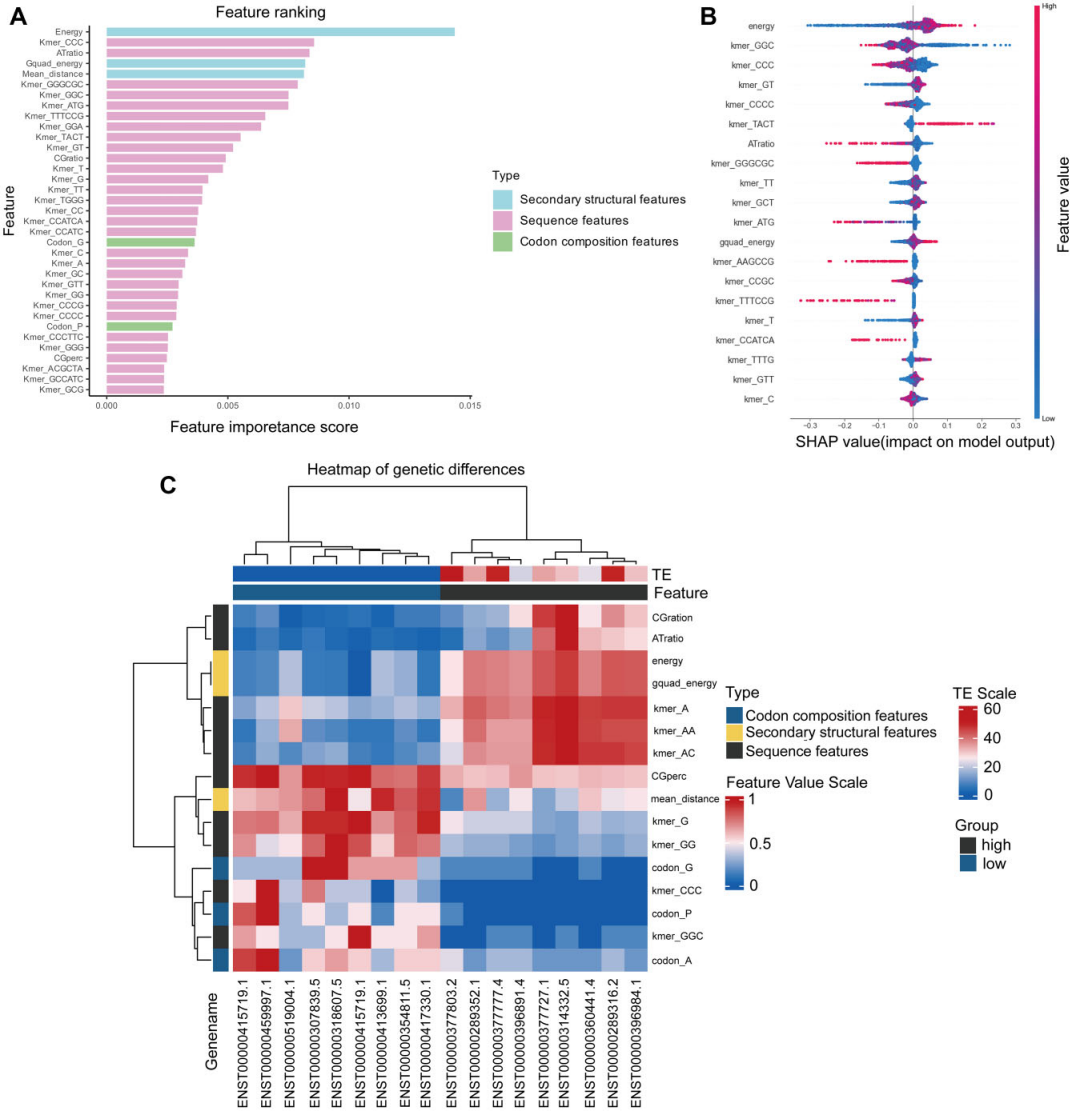
Model visualization and gene differential heatmap. (**A**) Feature ranking. Features were divided into three groups: sequence features, RNA secondary structure features, and codon usage frequency features. The top 35 features ranked by contribution were calculated using the feature importance function of the RF model. (**B**) Beeswarm plot of the top 20 features based on SHAP values in RF. In the beeswarm plot, the features are sorted by the sum of SHAP value magnitudes. The SHAP values are used to show the distribution of the impact on model prediction of each feature (positive values indicate positive effects, and negative values indicate negative effects). (**C**) Feature differential heatmap. The top 9 genes with the highest TE values and the bottom 9 genes with the lowest TE values were selected for differential feature analysis. Blue indicates lower values, while red indicates higher values.

To gain deeper insights into the determinants of TE, we performed extensive model interpretation analyses. Using the TE value data from the PC3 cell line (*n* = 1376) (Fig. 1E and Supplementary Table S1), we generated native importance maps, gene differential heatmaps, and SHAP (SHapley Additive exPlanations) plots to interrogate the patterns learned by both models. These visual tools allowed us to identify key sequence features and structural motifs correlated with higher TE, providing biological plausibility to our predictive models. We extracted a comprehensive set of features, including *k*-mer frequency [31], GC content, codon frequency [32–34], and RNA secondary structure metrics (MFE, mean_distance, and Gquad_energy) [35, 36], to assess their impact on TE. RF analysis revealed that RNA secondary structure dominated feature importance (Fig. 5A). Lower MFE values, indicative of higher RNA stability, were associated with reduced TE, likely because stable secondary structures hinder ribosome binding and scanning. This viewpoint is also clearly reflected in the SHAP beeswarm plot, where lower minimum free energy contributes significantly to the model’s negative predictions (Fig. 5B). Gquad_energy further refined this by accounting for G-quadruplexes [37–39], which are known to stabilize RNA structures and impede translation initiation. Additionally, CG-rich *k*-mers were identified as important features, potentially due to their role in forming stable secondary structures or CpG islands [40], which may indirectly influence TE by affecting transcriptional activity or mRNA stability. Consistent with this viewpoint, the SHAP beeswarm plot further demonstrated that CG-rich *k*-mer frequencies contributed negative predictive effects to the model, whereas AT-rich *k*-mer frequencies positively predicted TE values. To systematically evaluate the parametric contributions of different feature clusters, we performed ablation experiments by sequentially removing specific feature groups. The results demonstrated that the combination of *k*-mer features and RNA secondary structure metrics achieved the highest predictive performance (Supplementary Fig. S8), aligning with the dominance of RNA structural features observed in the RF importance analysis. While *k*-mer features constituted the primary compositional basis, RNA structural features dominated predictive capacity, underscoring their biological significance in ribosome binding and scanning dynamics.

Gene differential heatmap analysis (Fig. 5C) highlighted distinct feature preferences between high- and low-TE genes. High-TE genes exhibited higher MFE and Gquad_energy (indicative of weaker RNA stability), along with enrichment of A-rich *k*-mers that may facilitate ribosome access. In contrast, low-TE genes were characterized by higher CG content (averaging 0.6 compared to 0.4 in high-TE genes) and more stable secondary structures. The negative correlation between CG-rich *k*-mers and TE suggests these sequences may either form stable RNA structures impeding ribosome progression or associate with CpG islands [40, 41], where DNA methylation and transcriptional silencing could reduce mRNA availability. Furthermore, the shorter mean_distance observed in high-TE genes (∼20 bp versus 30 bp in low-TE genes) suggests a balance between structural flexibility and compactness, potentially optimizing ribosome binding while maintaining mRNA stability. These findings align with prior studies demonstrating that AT-rich sequences promote translation initiation, while CG-rich regions form stable structures hindering ribosome binding and scanning [13]. Together, these results underscore the interplay between RNA sequence, structure, and epigenetic regulation in shaping TE.

## Supplementary Material

gkaf861_Supplemental_File

## Data Availability

The data underlying this article are available in the Figshare Digital Repository. The RF model data can be accessed at https://doi.org/10.6084/m9.figshare.28681190 and the corresponding GitHub repository at https://github.com/fightw/5UTR_RFuploadw. The SeqNet model data are available at https://doi.org/10.6084/m9.figshare.28681223 and its GitHub implementation at https://github.com/fightw/modules_SeqNN.
