# Supplementary material for: Machine learning-based analysis of the impact of 5′ untranslated region on protein expression
Source: Nucleic Acids Res. 2025 Sep 9;53(17):gkaf861. doi: 10.1093/nar/gkaf861 (PMC12418383; doi:10.1093/nar/gkaf861)
Supplement: gkaf861_Supplemental_File [file gkaf861_supplemental_file.doc]

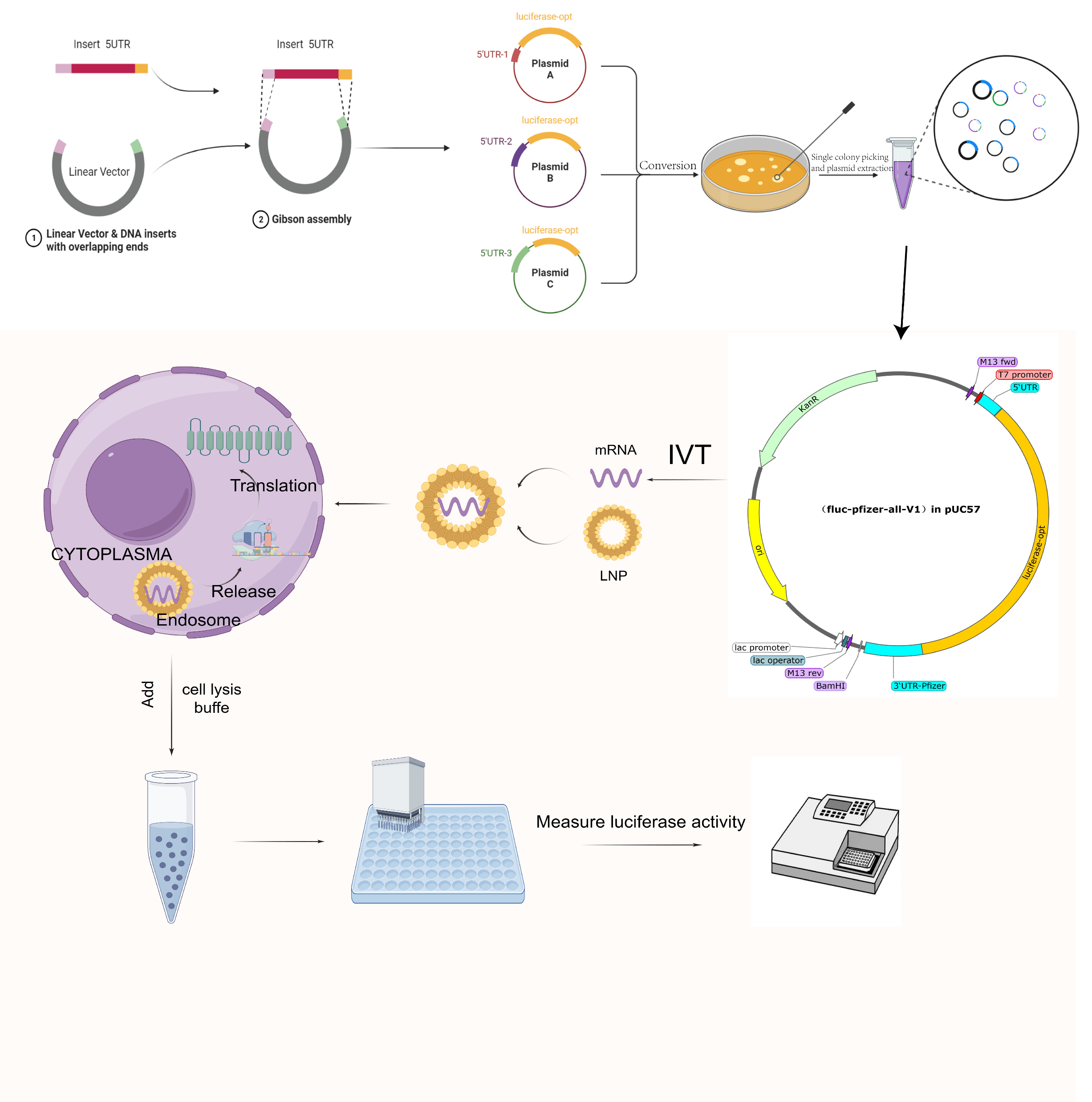


**Fig. S1.Schematic diagram of the experimental process.** The target fragment 5’UTR was ligated into the vector and transformed, monoclonalized and amplified. Plasmids were linearized, polyadenylated, capped with *in vitro* co-transcription, and encapsulated in lipid nanoparticles using an automated liquid handling system.


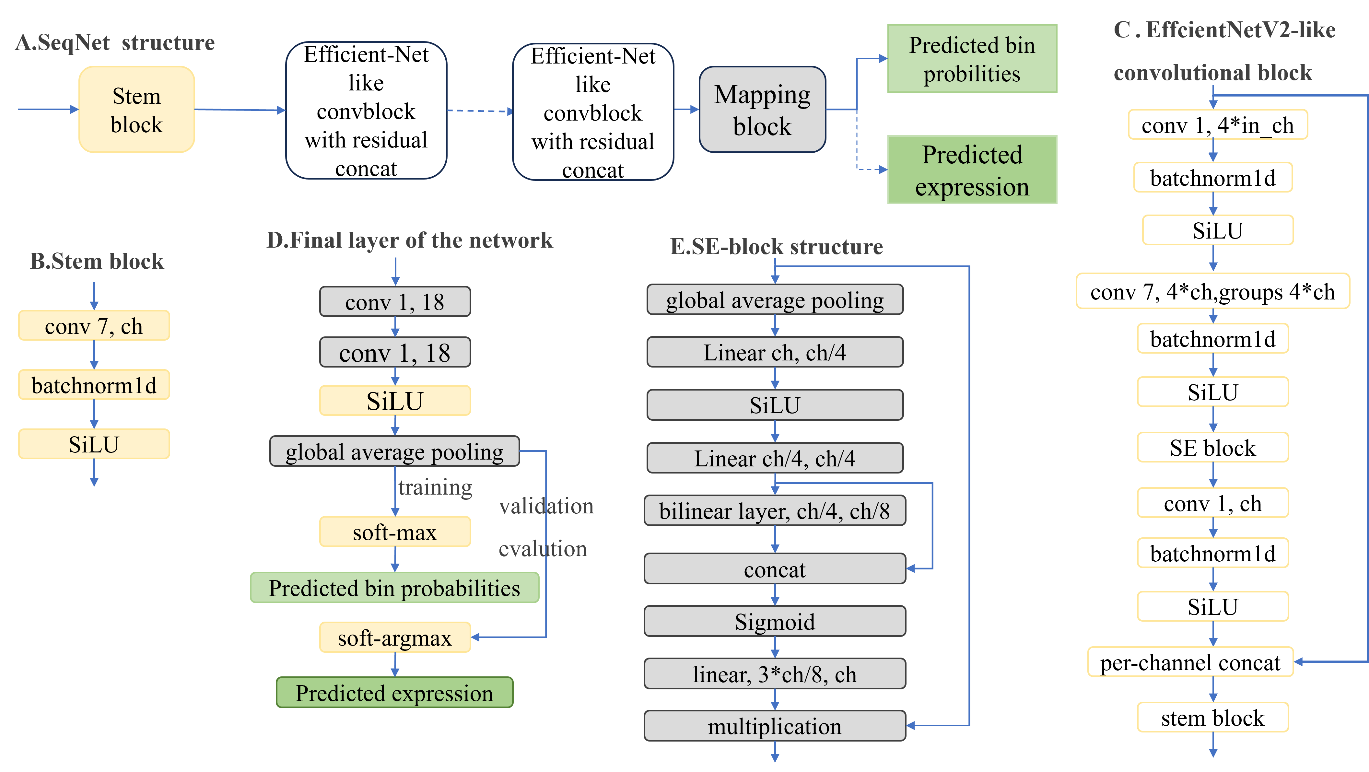


**Fig. S2. SeqNet model architecture schematic.** (A) Model overview. (B) Stem block structure, consisting of a standard convolutional layer, a batch normalization layer (BatchNorm), and a SiLU activation function layer. (C) EfficientNetV2-like convolutional block, with grouped convolutions (replacing depthwise convolutions) to balance efficiency and expressiveness, channel-wise residual connections for parameter efficiency, and "Same" padding applied uniformly across all convolutional layers. The channel progression includes six blocks with [128, 128, 64, 64, 64, 64] output channels, respectively. (D) Final layer of the network, including pointwise convolution (1×1 kernel) for channel dimension adjustment, channel-wise global average pooling to aggregate spatial features, and SoftMax activation for classification probabilities. (E) SE-block structure, incorporating low-rank canonical polyadic decomposition (via TensorLy) in the bilinear block to reduce parameter count, a squeeze operation (global average pooling to capture channel-wise statistics), and an excitation operation.

###
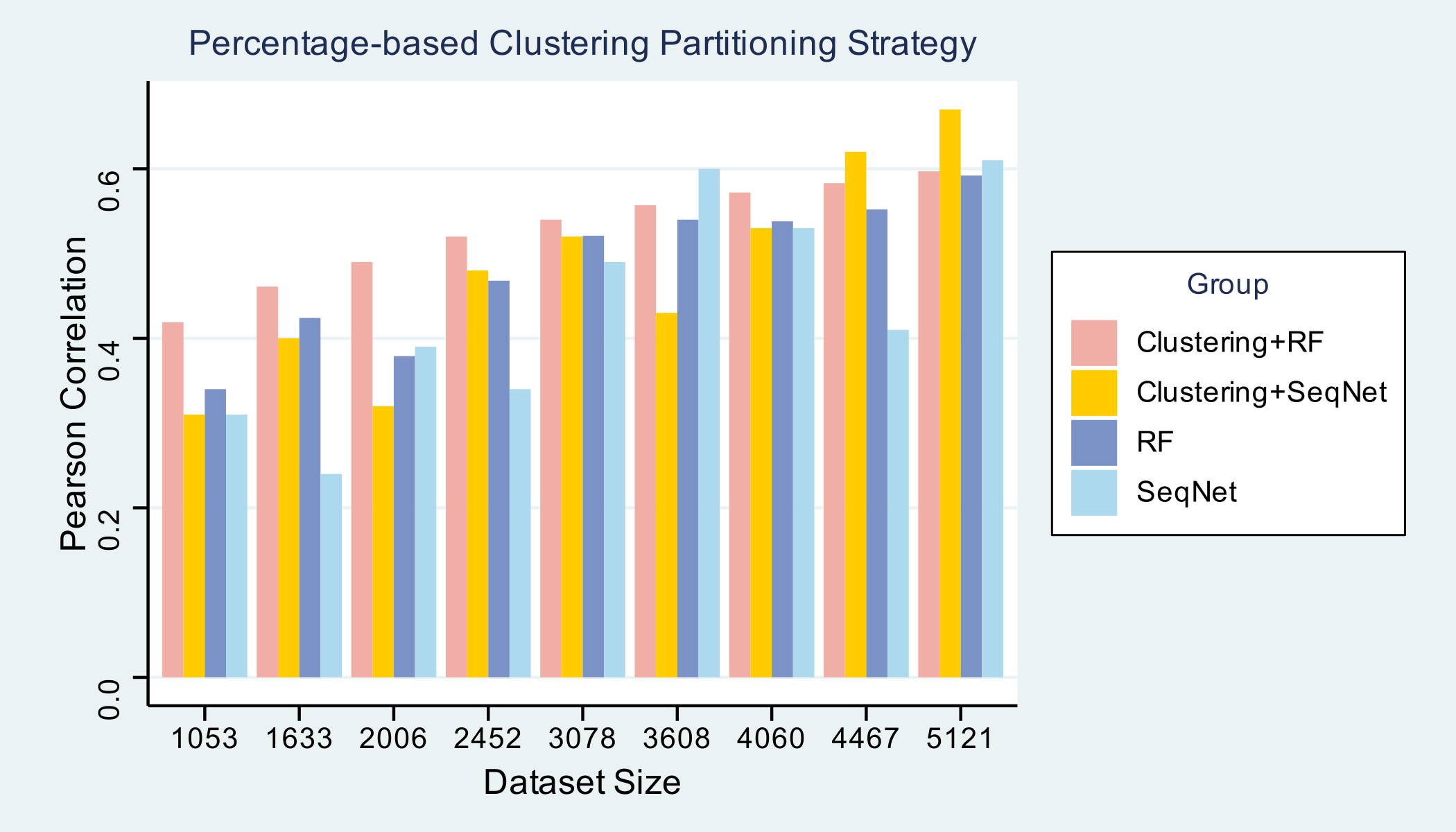


**Fig. S3.** The x-axis represents the dataset size, and the y-axis shows the Pearson correlation coefficient. The pink bar represents the Random Forest model trained on the clustered training set, the yellow bar represents the SeqNet model trained on the clustered training set, the dark blue bar represents the Random Forest model trained on the random training set, and the light blue bar represents the SeqNet model trained on the random training set .


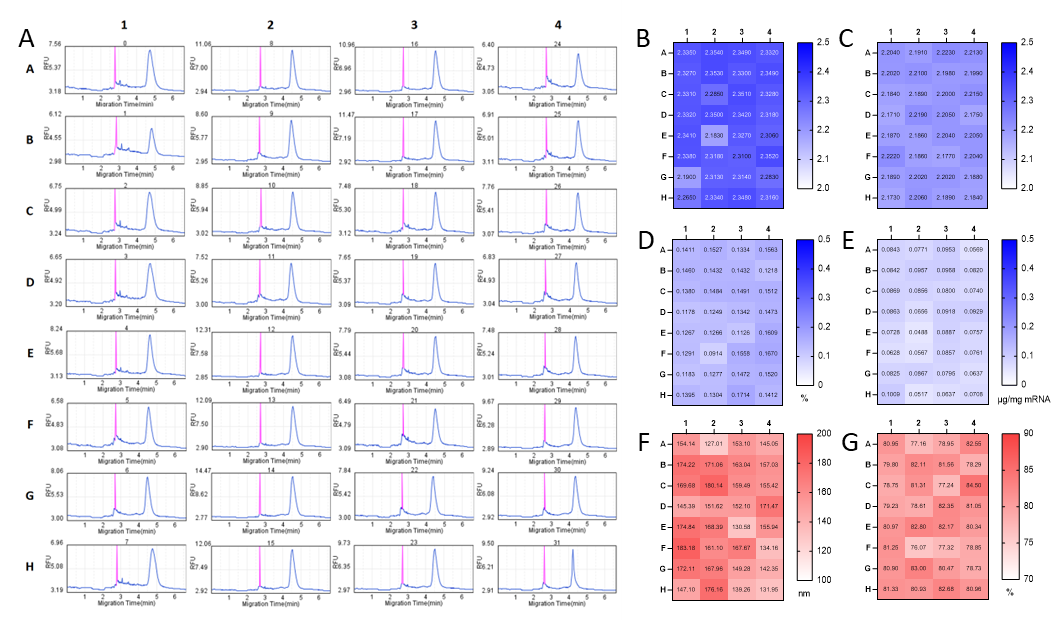


**Fig. S4. Representative quality control of mRNA and LNPs prepared in this study.** (A) Capillary electrophoresis analysis. (B) A260/A280 ratios of mRNAs. (C) A260/A230 ratios of mRNAs. (D, E) Quantification of residual dsRNA and T7 RNA polymerase in mRNA samples, respectively. (F, G) Particle size determination and encapsulation efficiency of LNPs.

**Fig. S5.** Average bioluminescence intensity of firefly luciferase mRNA containing representative 5’UTR upon transfection into HeLa and HEK293T cells. Data are shown as mean ± SD (n = 5 biologically independent samples).


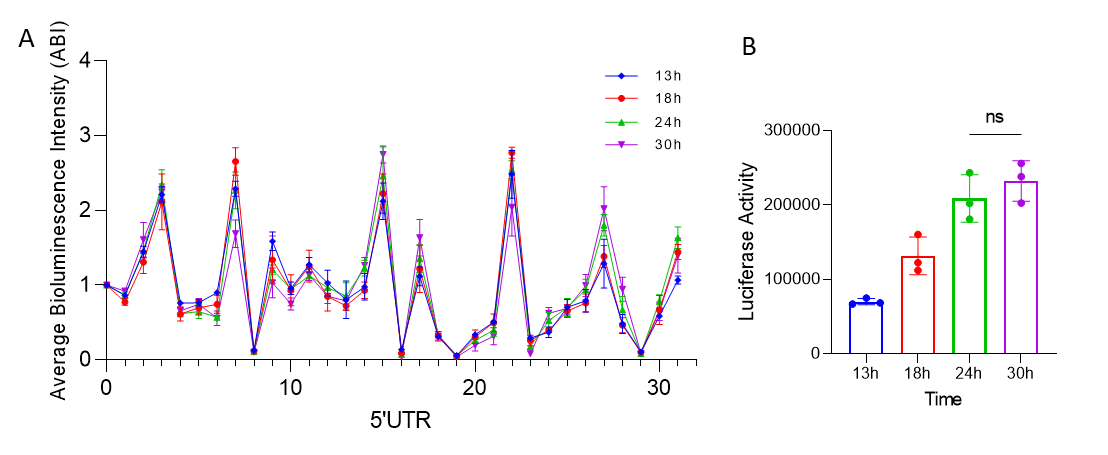


**Fig. S6.** (A) Average bioluminescence intensity of firefly luciferase mRNA containing a representative 5’UTR in HeLa cells at multiple time points (13, 18, 24, and 30 h post-transfection). (B) Luciferase activity of firefly luciferase mRNA containing the reference 5’UTR (5’UTR-0) in HeLa cells at the same time points. Data are shown as mean ± SD (n = 3 biologically independent samples for duplicate measurements). Statistical significance was analyzed by Tukey's multiple comparisons test. ns, not significant.


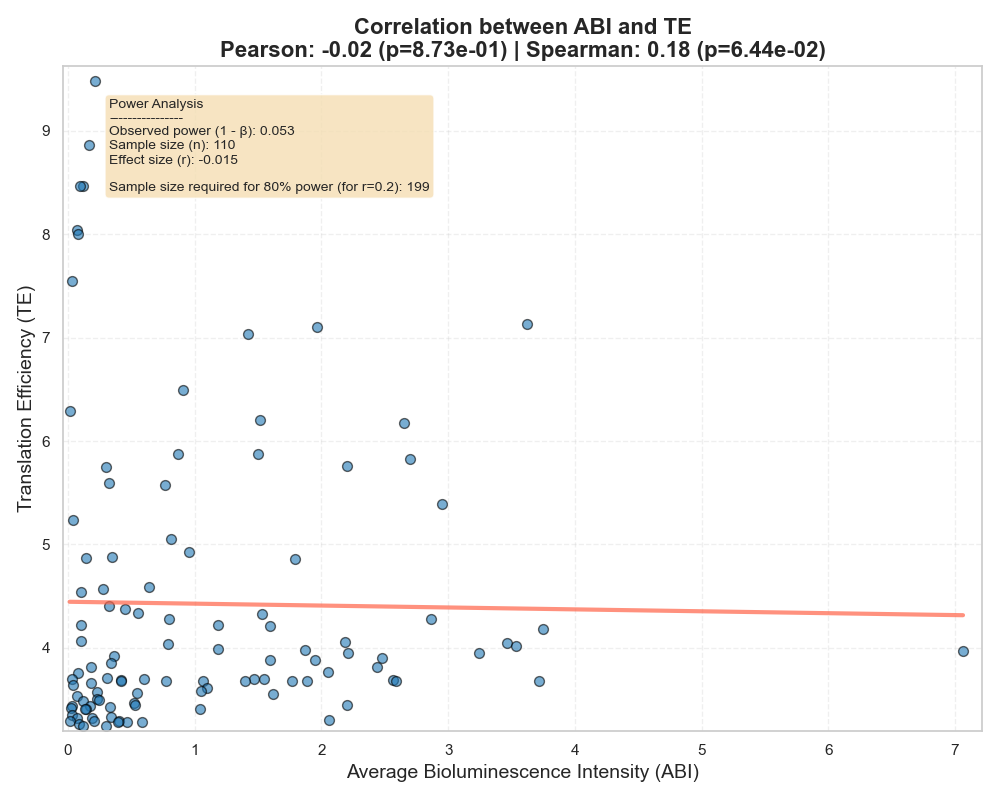

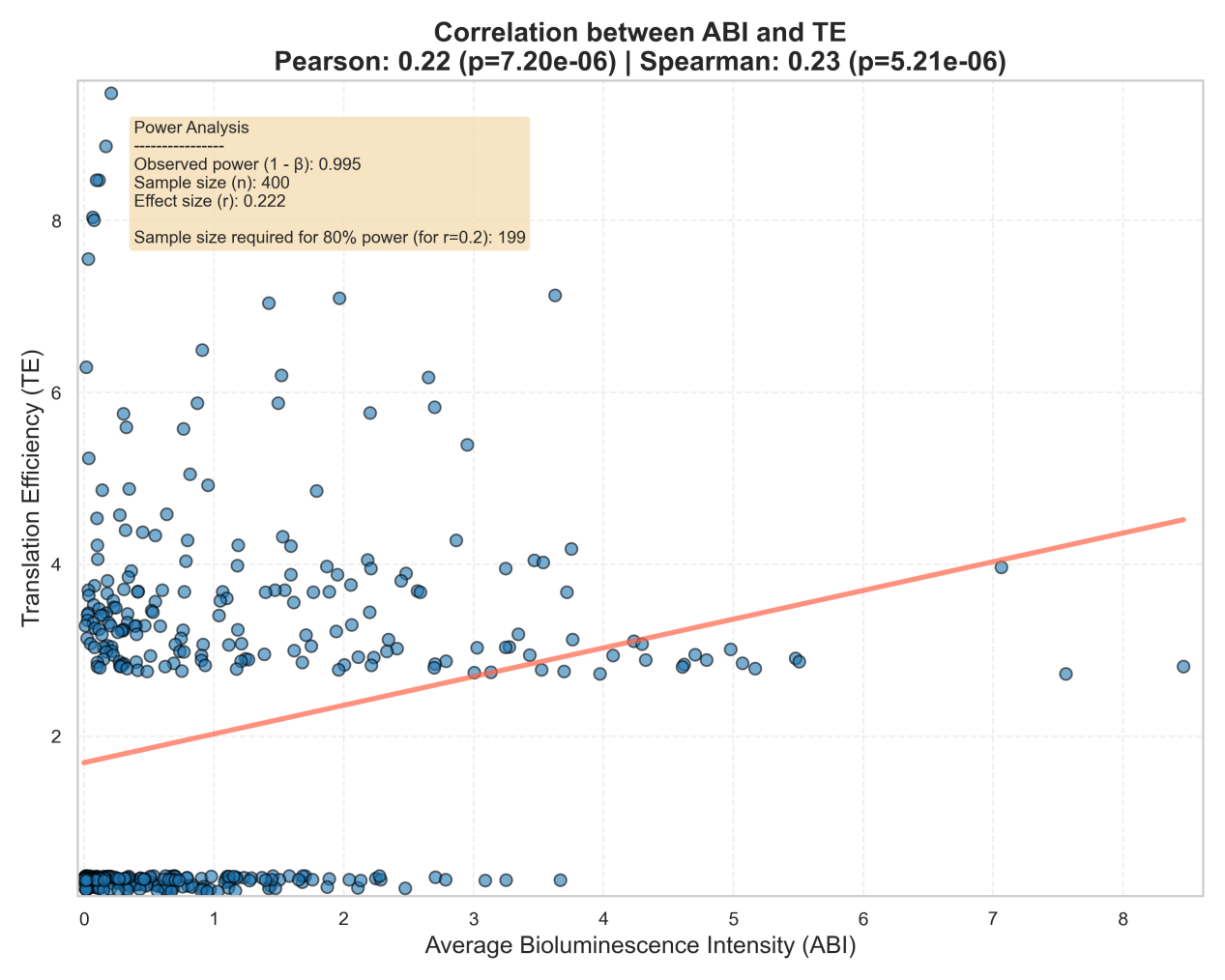


**Fig. S7. Power analysis.** The x-axis represents normalized average bioluminescence intensity from wet-lab experiments, and the y-axis represents annotated TE values. The yellow layer illustrates the power analysis results, where observed power (statistical probability of detecting a true effect based on actual data), sample size (number of experimental data points), and effect size (magnitude of the relationship between variables) are depicted. Left: Analysis with 110 data points. Right: Extended analysis with 400 data points. (Observed Power ≥ 80% indicates reliability, and Observed Power = 0.995 reflects extremely high confidence in the detected effect.)





**Fig. S8. Ablation experiment.** Feature set ablation study on the random forest model for TE tasks, including codon usage frequency (codon), RNA secondary structure features (RNA SS), K-mer (and other sequence feature modules).

**Table S1.** Summary of Datasets, Training Strategies, and Step-wise Performance

| **Process** | **DataSet** | **Training Strategy** | **Performance** |
| --- | --- | --- | --- |
| Data Collection | Three human datasets from cell lines（n=6721） | \ | \ |
| Model Training | Three human datasets from cell lines（n=6721） | tenfold ross-validation | \ |
| Model compare | Three human datasets from cell lines（n=6721） | tenfold ross-validation | PearsonR |
| Clustering | Three human datasets from cell lines（n=6721） | Cluster train set and specific test set | PearsonR/data compression rate /model accuracy restoration |
| Experimental Validation / Cross-Datase Validation | human datasets from PC3 cell lines（n=1376）/ human datasets（ABI） from PC3 cell lines（n=400） | Cluster train set and specific test set | PearsonR |
| Model isualization and Inference | human datasets from PC3 cell lines（n=1376） | \ | Feature_importance score/SHAP value/Feature values |

**Table S2.** Primers used in this manuscript

| Primer Name | Primer Sequence |
| --- | --- |
| Forward primer | 5ʹ>CTATGCGGCATCAGAGCAGA<3ʹ |
| Reverse primer | 5ʹ>TTTTTTTTTTTTTTTTTTTTTTTTTTTTTTTTTTTTTTTTTTTTTTTTTTTTTTTTTTTTTTTTTTTTTTTTTTTTTTTTTTTTTTTTTTTTTTTTTTTTTTTTTTTTTTTTTTTTTTTTAGTAACGGCAGACTTCTCCTG<3ʹ |
